# Predictive value of sarcopenia components for all-cause mortality: findings from population-based cohorts

**DOI:** 10.1007/s40520-024-02783-x

**Published:** 2024-06-06

**Authors:** Leo D. Westbury, Nicholas C. Harvey, Charlotte Beaudart, Olivier Bruyère, Jane A. Cauley, Peggy Cawthon, Alfonso J. Cruz-Jentoft, Elizabeth M. Curtis, Kristine Ensrud, Roger A. Fielding, Helena Johansson, John A. Kanis, Magnus K. Karlsson, Nancy E. Lane, Laetitia Lengelé, Mattias Lorentzon, Eugene McCloskey, Dan Mellström, Anne B. Newman, Claes Ohlsson, Eric Orwoll, Jean-Yves Reginster, Eva Ribom, Björn E. Rosengren, John T. Schousboe, Elaine M. Dennison, Cyrus Cooper, and the International Musculoskeletal Ageing Network

**Affiliations:** 1 MRC Lifecourse Epidemiology Centre, University of Southampton, Southampton, UK; 2 NIHR Southampton Biomedical Research Centre, University of Southampton and University Hospital Southampton NHS Foundation Trust, Southampton, UK; 3 Department of Biomedical Sciences, Clinical Pharmacology and Toxicology Research Unit, Namur Research Institute for Life Sciences (NARILIS), Faculty of Medicine, University of Namur, 5000 Namur, Belgium; 4 Division of Epidemiology, Public Health and Health Economics, Department of Public Health, University of Liège, Liège, Belgium; 5 Department of Epidemiology, School of Public Health, University of Pittsburgh, Pittsburgh, PA USA; 6 Research Institute, California Pacific Medical Center, San Francisco, California USA; 7 Department of Epidemiology and Biostatistics, University of California, San Francisco, CA USA; 8 Servicio de Geriatría, Hospital Universitario Ramón y Cajal (IRYCIS), Madrid, Spain; 9 Medicine and Epidemiology & Community Health, University of Minnesota, Minnesota, USA; 10 Center for Care Delivery and Outcomes Research, Minneapolis VA Health Care System, Minneapolis, MN USA; 11 Nutrition, Exercise Physiology, and Sarcopenia Laboratory, Jean Mayer USDA Human Nutrition Research Center On Aging, Tufts University, Boston, USA; 12 Mary MacKillop Institute for Health Research, Australian Catholic University, Melbourne, Australia; 13 Sahlgrenska Osteoporosis Centre, Institute of Medicine, University of Gothenburg, Gothenburg, Sweden; 14 Centre for Metabolic Bone Diseases, University of Sheffield, Sheffield, UK; 15 Clinical and Molecular Osteoporosis Research Unit, Department of Clinical Sciences Malmo, Lund University and Department of Orthopedics, Skane University Hospital, Malmo, Sweden; 16 Division of Rheumatology, Department of Internal Medicine, UC Davis Health, 4625 Second Avenue, Sacramento, CA 95917 USA; 17 Metabolism and Nutrition Research Group, Louvain Drug Research Institute, UCLouvain, Université catholique de Louvain, 1200 Sint-Lambrechts-Woluwe, Belgium; 18 Center for Osteoporosis Research, Institute of Medicine, Sahlgrenska Academy, Sahlgrenska University Hospital, Mölndal, Sweden; 19 Centre for Integrated Research in Musculoskeletal Ageing (CIMA), Mellanby Centre for Bone Research, University of Sheffield, Sheffield, UK; 20 Centre for Bone and Arthritis Research (CBAR), Sahlgrenska Academy, University of Gothenburg, Gothenburg, Sweden; 21 Department of Internal Medicine and Clinical Nutrition, Institute of Medicine, Sahlgrenska Osteoporosis Centre, Centre for Bone and Arthritis Research at the Sahlgrenska Academy, University of Gothenburg, Gothenburg, Sweden; 22 Region Västra Götaland, Sahlgrenska University Hospital, Department of Drug Treatment, Gothenburg, Sweden; 23 Oregon Health & Science University, Portland, Oregon USA; 24 Protein Research Chair, Biochemistry Department, College of Science, King Saud University, Riyadh, Kingdom of Saudi Arabia; 25 Department of Surgical Sciences, University of Uppsala, Uppsala, Sweden; 26 Park Nicollet Clinic and HealthPartners Institute, Bloomington, Minnesota USA; 27 University of Minnesota, Minneapolis, Minnesota USA; 28 Victoria University of Wellington, Wellington, New Zealand; 29 NIHR Oxford Biomedical Research Centre, University of Oxford, Oxford, UK

**Keywords:** Epidemiology, Osteoporosis, Sarcopenia, Ageing, Mortality

## Abstract

**Background:**

Low grip strength and gait speed are associated with mortality. However, investigation of the additional mortality risk explained by these measures, over and above other factors, is limited.

**Aim:**

We examined whether grip strength and gait speed improve discriminative capacity for mortality over and above more readily obtainable clinical risk factors.

**Methods:**

Participants from the Health, Aging and Body Composition Study, Osteoporotic Fractures in Men Study, and the Hertfordshire Cohort Study were analysed. Appendicular lean mass (ALM) was ascertained using DXA; muscle strength by grip dynamometry; and usual gait speed over 2.4–6 m. Verified deaths were recorded. Associations between sarcopenia components and mortality were examined using Cox regression with cohort as a random effect; discriminative capacity was assessed using Harrell’s Concordance Index (C-index).

**Results:**

Mean (SD) age of participants (*n* = 8362) was 73.8(5.1) years; 5231(62.6%) died during a median follow-up time of 13.3 years. Grip strength (hazard ratio (95% CI) per SD decrease: 1.14 (1.10,1.19)) and gait speed (1.21 (1.17,1.26)), but not ALM index (1.01 (0.95,1.06)), were associated with mortality in mutually-adjusted models after accounting for age, sex, BMI, smoking status, alcohol consumption, physical activity, ethnicity, education, history of fractures and falls, femoral neck bone mineral density (BMD), self-rated health, cognitive function and number of comorbidities. However, a model containing only age and sex as exposures gave a C-index (95% CI) of 0.65(0.64,0.66), which only increased to 0.67(0.67,0.68) after inclusion of grip strength and gait speed.

**Conclusions:**

Grip strength and gait speed may generate only modest adjunctive risk information for mortality compared with other more readily obtainable risk factors.

**Supplementary Information:**

The online version contains supplementary material available at 10.1007/s40520-024-02783-x.

## Background

Sarcopenia is characterised by the excessive loss of muscle mass, strength and function with advancing age. Consequences of sarcopenia include increased risk of frailty and earlier mortality, significant loss of quality of life and considerable healthcare expenditure [1–4]. Sarcopenia has been recognised as a medical condition since 2016 according to the International Classification of Diseases [5].

Most sarcopenia definitions incorporate measures of grip strength, gait speed or lean mass. However, research over the last decade has demonstrated greater capacity of grip strength and gait speed to predict incident adverse health outcomes in comparison with measures of lean mass, particularly appendicular lean mass from dual-energy X-ray absorptiometry (DXA) [6]. Indeed, more recent sarcopenia definitions proposed by the 2019 European Working Group on Sarcopenia in Older People (EWGSOP2) [7] and the Sarcopenia Definitions and Outcomes Consortium (SDOC) [8] either place less importance on lean mass (EWGSOP2) or do not include this measure in the algorithm (SDOC).

Many studies have examined sarcopenia components in relation to risk of earlier mortality. However, to date, there is limited information on how much these measures might add, in terms of outcome prediction, to the risk information associated with clinical risk factors such as age, sex, BMI and smoking status, which are known to strongly influence mortality risk and can be easily ascertained from routine clinical data. We therefore aimed to quantify the additional predictive value of grip strength and gait speed, over and above other clinical risk factors in predicting mortality, using data from a multinational assembly of cohort studies comprising the Health, Aging and Body Composition (Health ABC) Study (USA), Osteoporotic Fractures in Men (MrOS) Study (USA) and the UK-based Hertfordshire Cohort Study (HCS).

## Methods

### Cohort studies

The Health ABC Study consists of 3075 US men and women (aged 70–79 years), who were recruited in 1997 to 1998 [9]. A random sample of White ethnicity and Black ethnicity Medicare beneficiaries from around Pittsburgh and Memphis was ascertained. Selected participants received a mailing and then a telephone eligibility screen. Participants reporting no difficulty in ascending 10 stairs or walking one quarter of a mile were eligible. The exclusion criteria were as follows: intending to move outside the area within three years; currently enrolled in a lifestyle intervention study; unable to communicate with the interviewer; clear cognitive impairment; having difficulties with activities of daily living or having a life-threatening illness; or requiring a walking aid. Institutional review boards at the University of Pittsburgh and the University of Tennessee approved the study. All participants provided written informed consent.

MrOS US comprises 5994 men (aged 65–100) who were enrolled from March 2000 to April 2002 at six sites [10, 11]. The following recruitment methods were utilised: targeted presentations; advertisements and features in seniors’ newspapers; participant and voter registration databases; and mailings from the Department of Motor Vehicles [12]. Only those without bilateral hip replacements and who could walk without assistance were eligible. Self-reported ethnicity was recorded. The study was approved by institutional review boards at each site. Written informed consent was provided by all participants.

The HCS consists of 2997 men and women born in Hertfordshire (UK) from 1931 to 1939 and who still lived there in 1998–2004, when they attended a baseline home interview and research clinic for a health assessment. Further details about this study have been published previously [13, 14]. A subset of HCS participants who underwent whole body DXA during a follow-up study in 2011 to 2012 (*n* = 346) were the basis of the HCS analysis in this manuscript. The Hertfordshire and Bedfordshire Local Research Ethics Committee provided approval for the baseline home interview and research clinic; all HCS follow-up studies also had ethical approval. All participants gave written informed consent.

### Ascertainment of participant characteristics

Details on the ascertainment of participant information, including the procedures and measurement devices used, are provided in Table 1.

**Table 1:** Ascertainment of participant information within each cohort

Participant characteristic	Osteoporotic Fractures in Men (MrOS) US Study	Health, Aging and Body Composition (Health ABC) Study	Hertfordshire Cohort Study (HCS)
Height	Measured using a Harpenden stadiometer		
Weight	Measured using an electric scale or balance beam scale	Measured using a standard balance beam scale	Measured using a SECA floor scale, Chasmors Ltd, London, UK
**Ascertained through researcher-administered questionnaires**			
Current smoker	Categorised as ‘current smoker’ or ‘never/previous smoker’		
High alcohol consumption	>7 drinks per week	>1 drink per day	>14 units per week
Physical activity	Assessed using the Physical Activity Scale for the Elderly [41]	Assessed over the past 7 days; approximate metabolic equivalent unit values were assigned to reported activities and intensity levels to derive caloric expenditure [42]. Total kilocalories expended per week was calculated as previously described [43].	Assessed using the Longitudinal Aging Study Amsterdam Physical Activity Questionnaire [44]
Ethnicity	Ethnicity was self-reported and categorised as ‘White’ and ‘BAME’ for this analysis	White and black participants were recruited; black participants were categorised as ‘BAME’	All participants were white
Left school early	Whether participants completed high school or not was ascertained from highest level of education attained	Whether participants completed Grade 12 or not was ascertained from highest level of education attained	Whether participants left school before 15 years of age or not was ascertained from age of leaving full-time education
Fall in previous year	Falls in previous 12 months were self-reported		
Prior fracture	Fractures since age 50 years were self-reported	Fractures since age 45 years were self-reported	
Self-rated health (<good)	Self-rated health was ascertained from five multiple choice options and dichotomised as ‘good or better’ or ‘less than good’		
Low cognitive function	Obtained from the Modified Mini-Mental State Examination (3MS) [45] and categorised as <80 or ≥80 as in previous analyses of the MrOS US Study [46, 47] and the Health ABC Study [48, 49]		Obtained from the Mini-Mental State Examination (MMSE) [50] and categorised as <24 or ≥24 as in many previous studies [51]
Number of comorbidities	Calculated from the number of the following doctor-diagnosed comorbidities that were self-reported:		
	MrOS US • Heart disease (congestive heart failure, myocardial infarction, angina) • Lung disease (COPD) • Hypertension • Diabetes • Stroke • Arthritis • Osteoporosis • Thyroid disease • Parkinson's • Cancer	Health ABC • Heart disease (congestive heart failure, myocardial infarction, angina) • Lung disease (COPD, asthma, pneumonia) • Hypertension • Diabetes • Stroke • Arthritis • Osteoporosis • Thyroid disease • Parkinson's • Cancer	HCS • Heart disease (heart failure, myocardial infarction, angina) • Lung disease (COPD, asthma) • Hypertension • Diabetes • Stroke • Rheumatoid arthritis • Osteoporosis • Thyroid disease • Parkinson's • Cancer
Gait speed (m/s)	Calculated from the fastest time from two 6m gait speed tests. Participants were asked to walk at their usual pace.		Calculated from the fastest time from two 2.44m (8ft) gait speed tests. Participants were asked to walk at their usual pace.
Grip strength (kg)	Assessed twice for each hand using a Jamar dynamometer; the highest measurement was used for analysis. Participants with recent arthritis/pain in their wrist or hand or who had undergone surgery of the upper extremity in the past 3 months did not have their grip strength assessed on that side.		Assessed three times for each hand using a Jamar dynamometer; the highest measurement was used for analysis
ALM index (kg/m^2^)	ALM was ascertained from whole-body dual-energy X-ray absorptiometry scans (Hologic QDR 4500 [Hologic, Bedford, MA, USA])	ALM was ascertained from whole-body dual-energy X-ray absorptiometry scans (Hologic QDR 4500A; Hologic, Bedford, MA, USA)	ALM was ascertained from whole-body dual-energy X-ray absorptiometry scans (Lunar Prodigy Advanced Scanner, GE Medical Systems, UK)
Femoral neck BMD (g/cm^2^)	Ascertained by DXA using the same device as used in each cohort for measurement of ALM. T-scores were derived using US National Health and Nutrition Examination Survey (NHANES) III White female reference data from 20 to 29 year-olds [17], as recommended by the International Society for Clinical Densitometry (ISCD).		
Mortality	Deaths were centrally adjudicated by physician review of death certificates and additional medical records	Deaths were determined from death certificates, hospital records and interviews with next of kin. All deaths were adjudicated by a central committee.	This cohort was flagged on the NHS Central Register for continuous notification of deaths

*BAME* Black, Asian and minority ethnic; *ALM* Appendicular lean mass

### Statistical methods

Summary statistics were used to describe participant characteristics. Cox regression models with cohort as the shared statistical frailty factor and mortality as the outcome were implemented; the shared frailties, assumed to be gamma-distributed latent random effects, reflect the fact that participants from the same cohort are likely to have more similar risks of mortality than participants from different cohorts. Different sets of exposures were defined as follows: Set 1: age and sex; Set 2: Set 1 + BMI, current smoker (yes/no), high alcohol consumption (yes/no), prior fracture since age 45 years (50 years in MrOS US Study) (yes/no), and femoral neck BMD T-score; Set 3: Set 2 + physical activity, BAME (Black, Asian and minority ethnic) ethnicity (yes/no), left school early (yes/no), fall in previous 12 months (yes/no), self-rated health of less than good (yes/no), low cognitive function (yes/no), and number of comorbidities. Set 2 comprised some of the key risk factors used in FRAX, the fracture risk assessment tool [15] and included BMD which is typically derived in the process used to ascertain ALM; relationships between lower BMD and increased mortality risk have also been reported in the literature, although this association may not be causal [16]. The following Cox models were then implemented: linear combinations of ALM index, grip strength and gait speed as exposures; Sets 1–3 as exposures; Sets 1–3 as exposures in addition to ALM index, grip strength and gait speed. For each model, the discriminative capacity according to Harrell’s Concordance Index (C-index) was examined as well as the strength of association between each sarcopenia component included in the model and risk of mortality. The C-index estimates the probability that for a randomly selected pair of participants, the participant with the higher predicted risk of the outcome experiences the outcome earlier. It ranges from 0 to 1 with 0.5 corresponding to the performance of a random classifier.

Cox models were then implemented to examine the relationship between each exposure and death with adjustment for age and sex; statistically significant exposures were then included in a single mutually-adjusted model. Each model was evaluated using the C-index. A minimal model, based on a small number of easily obtainable exposures, was then developed with the aim of achieving a similar C-index as the mutually-adjusted model.

Contour plots were produced showing the 5-year probability of death according to grip strength and gait speed. These were estimated at 25th, 50th and 75th centiles (sex-specific) of ALM index and at the mean age (based on the entire sample of participants). Contour plots were estimated separately among men and women using logistic regression models with ALM index, grip strength, gait speed and age as linear terms.

Physical activity was assessed differently across cohorts so values were standardised within each cohort; femoral neck BMD T-scores were derived using US National Health and Nutrition Examination Survey (NHANES) III White female reference data from 20 to 29 year-olds [17]); and the remaining continuous measures were assessed on the same scale and, therefore, were standardised among the whole analysis sample. Analyses were conducted using Stata, release 17.0; men and women were pooled together in Cox models as sex-interactions regarding each sarcopenia component were not statistically significant (*p* > 0.05). The analytical sample comprised participants with complete data regarding all the variables used in the analysis.

### Sensitivity analyses

Cox regression analyses were conducted separately within each cohort as a sensitivity analysis to check that findings were not affected by pooling data across cohorts. Furthermore, for participants who did not have their gait speed assessed over 6 m (gait speed was assessed over 8ft in HCS), analyses were repeated when gait speed values in this cohort were converted to those expected over 6 m using previously published equations [18, 19]. The results presented below are based on the raw gait speed values.

## Results

### Descriptive statistics

Participant characteristics of the entire sample (*n* = 8362) and stratified by both sex and cohort are presented in Table 2. Mean age of the analysis sample was 73.8 (5.1) years. Overall, 5231 (62.6%) participants died during follow-up and 897 (10.7%) died within the first 5-years of follow-up; median (lower quartile, upper quartile) follow-up time to death or until participants were censored was 13.3 (8.0, 17.0) years.

**Table 2:** Participant characteristics stratified by cohort and sex

Participant characteristic	All cohorts (*n* = 8362)	MrOS US	Health ABC		HCS	
		Men (*n* = 5550)	Men (*n* = 1264)	Women (*n* = 1277)	Men (*n* = 139)	Women (*n* = 132)
Age (years)	73.8 (5.1)	73.6 (5.9)	74.2 (2.9)	74.0 (2.8)	75.2 (2.5)	75.4 (2.5)
BMI (kg/m^2^)	27.4 (4.1)	27.4 (3.8)	27.1 (3.9)	27.7 (5.4)	27.6 (3.7)	28.0 (4.5)
Current smoker	458 (5.5%)	194 (3.5%)	130 (10.3%)	124 (9.7%)	5 (3.6%)	5 (3.8%)
High alcohol consumption^a^	1194 (14.3%)	955 (17.2%)	156 (12.3%)	46 (3.6%)	34 (24.5%)	3 (2.3%)
Physical activity^b^	N/A	142.4 (100.8, 186.3)	5.5 (3.0, 8.9)	4.5 (2.7, 7.3)	193.6 (127.1, 285.7)	206.4 (146.8, 283.6)
Ethnicity (BAME)	1612 (19.3%)	585 (10.5%)	449 (35.5%)	578 (45.3%)	0 (0.0%)	0 (0.0%)
Left school early^c^	1003 (12.0%)	352 (6.3%)	333 (26.3%)	272 (21.3%)	24 (17.3%)	22 (16.7%)
Fall in previous year	1766 (21.1%)	1165 (21.0%)	226 (17.9%)	308 (24.1%)	34 (24.5%)	33 (25.0%)
Fracture since age 45 years^d^	1899 (22.7%)	1267 (22.8%)	209 (16.5%)	360 (28.2%)	29 (20.9%)	34 (25.8%)
Self-rated health (< good)	1177 (14.1%)	758 (13.7%)	193 (15.3%)	179 (14.0%)	22 (15.8%)	25 (18.9%)
Low cognitive function^e^	399 (4.8%)	146 (2.6%)	136 (10.8%)	95 (7.4%)	9 (6.5%)	13 (9.8%)
Number of comorbidities^f^	2.0 (1.0, 3.0)	2.0 (1.0, 3.0)	2.0 (1.0, 3.0)	2.0 (1.0, 3.0)	1.0 (1.0, 2.0)	1.0 (0.0, 2.0)
Gait speed (m/s)	1.22 (0.25)	1.25 (0.24)	1.24 (0.23)	1.13 (0.22)	0.82 (0.18)	0.77 (0.18)
Grip strength (kg)	38.6 (10.2)	41.7 (8.5)	40.9 (8.2)	25.0 (5.7)	37.4 (7.0)	22.0 (6.3)
ALM index (kg/m^2^)	7.7 (1.1)	8.0 (0.9)	8.0 (1.0)	6.5 (1.1)	8.1 (0.7)	6.4 (0.7)
Femoral neck BMD (g/cm^2^)	0.78 (0.14)	0.79 (0.13)	0.79 (0.14)	0.70 (0.13)	0.94 (0.13)	0.83 (0.12)

*MrOS* Osteoporotic Fractures in Men Study, *Health ABC* Health, Aging and Body Composition Study, *HCS* Hertfordshire Cohort Study, *BAME*: Black, Asian and minority ethnic, *ALM* Appendicular lean mass, *BMD* Bone mineral density

^a^ MrOS US (> 7 drinks per week); Health ABC (> 1 drink per day); HCS (> 14 units per week)

^b^ MrOS US (Physical Activity Scale for the Elderly score [possible range: 0–793]); Health ABC (Mcal/week); HCS (mins/day). Unable to present statistics for the entire cohort as units differ

^c^ MrOS US (did not complete high school); Health ABC (did not complete Grade 12); HCS (left school before age 15 years)

^d^ MrOS US (fracture since age 50 years)

^e^ MrOS US and Health ABC (Modified Mini-Mental State Exam score < 80); HCS (Mini-Mental State Exam score < 24)

^f^ Out of the following: heart disease, lung disease, hypertension, diabetes, stroke, arthritis, osteoporosis, thyroid disease, Parkinson’s and cancer

### Associations between sarcopenia components and mortality

Associations between sarcopenia components and mortality risk are presented in Table 3; hazard ratios for all exposures in the models are included in Supplementary Table 1. Lower ALM index, grip strength and gait speed were associated with increased mortality risk in univariate analysis. However, when these factors were included as exposures simultaneously, only grip strength (hazard ratio (95% CI) per SD reduction: 1.18 (1.14,1.23)) and gait speed (1.45 (1.40,1.49)) were associated (*p* < 0.05) with death, with much weaker associations observed for ALM index (1.02 (0.99,1.06), *p* = 0.135). Associations for grip strength and gait speed were similar in mutually-adjusted analysis which also included ALM index and key clinical risk factors (age, sex, BMI, smoking status, alcohol consumption, fracture history and femoral neck BMD T-score) as covariates: grip strength (hazard ratio per SD reduction: 1.18 (1.13,1.23)) and gait speed (1.31 (1.26,1.35)). These estimates were only slightly attenuated (grip strength 1.14 (1.10,1.19), gait speed 1.21 (1.17,1.26)) in the fully-adjusted model that also included physical activity, ethnicity, education, fall history, self-rated health, cognitive function and number of comorbidities. P-values for sex-interactions regarding ALM index, grip strength and gait speed were 0.072, 0.054 and 0.505 respectively.

**Table 3:** Mortality associations for sarcopenia components and discriminative capacity of models, depending on exposures included

Exposures included	C-index (95% CI)	Associations for sarcopenia components (per SD lower level of component)					
		ALM index (z-score)		Grip strength (z-score)		Gait speed (z-score)	
		HR (95% CI)	*P* value	HR (95% CI)	*P* value	HR (95% CI)	*P* value
ALM index	0.53 (0.53,0.54)	1.09 (1.06,1.12)	< 0.001	–	–	–	–
Grip strength	0.58 (0.57,0.58)	–	–	1.31 (1.27,1.35)	< 0.001	–	–
Gait speed	0.61 (0.60,0.61)	–	–	–	–	1.50 (1.46,1.55)	< 0.001
ALM index, grip strength	0.57 (0.57,0.58)	0.97 (0.94,1.01)	0.109	1.33 (1.28,1.37)	< 0.001	–	–
ALM index, gait speed	0.61 (0.60,0.62)	1.09 (1.06,1.12)	< 0.001	–	–	1.51 (1.46,1.55)	< 0.001
Grip strength, gait speed	0.61 (0.61,0.62)	–	–	1.20 (1.16,1.24)	< 0.001	1.44 (1.40,1.49)	< 0.001
ALM index, grip strength, gait speed	0.61 (0.61,0.62)	1.02 (0.99,1.06)	0.135	1.18 (1.14,1.23)	< 0.001	1.45 (1.40,1.49)	< 0.001
ALM index, grip strength, gait speed, Set 1	0.67 (0.67,0.68)	1.01 (0.98,1.05)	0.483	1.18 (1.14,1.23)	< 0.001	1.32 (1.28,1.37)	< 0.001
ALM index, grip strength, gait speed, Set 2	0.68 (0.67,0.69)	1.03 (0.98,1.09)	0.216	1.18 (1.13,1.23)	< 0.001	1.31 (1.26,1.35)	< 0.001
ALM index, grip strength, gait speed, Set 3	0.70 (0.69,0.70)	1.01 (0.95,1.06)	0.817	1.14 (1.10,1.19)	< 0.001	1.21 (1.17,1.26)	< 0.001
Set 1	0.65 (0.64,0.66)	–	–	–	–	–	–
Set 2	0.66 (0.65,0.67)	–	–	–	–	–	–
Set 3	0.69 (0.68,0.70)	–	–	–	–	–	–

*HR* Hazard ratio, *C-index* Harrell’s Concordance Index

Exposures included in each adjustment set:

Set 1: Age, sex

Set 2: Set 1, BMI, current smoker (yes/no), high alcohol consumption (yes/no), fracture since age 45 years (50 years in MrOS US Study) (yes/no), femoral neck BMD T-score

Set 3: Set 2, physical activity, BAME ethnicity (yes/no), left school early (yes/no), fall in previous 12 months (yes/no), self-rated health of less than good (yes/no), low cognitive function (yes/no), number of comorbidities

**Fig. 1:**
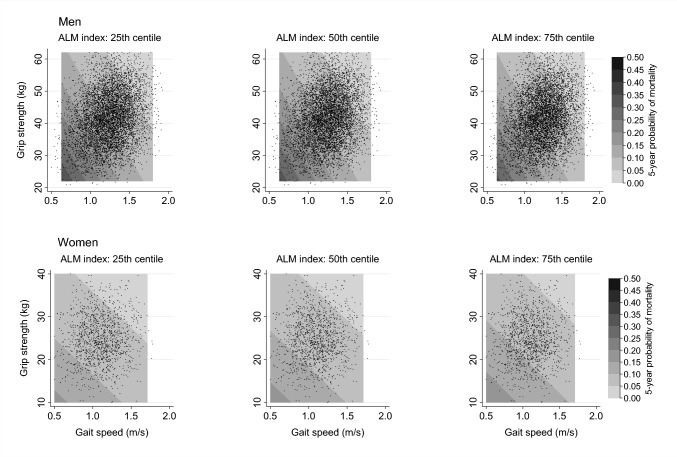
Probability of mortality within 5-years of follow-up according to ALM index, grip strength and gait speed. *ALM* Appendicular lean mass. Contour plots were estimated separately among men and women using logistic regression models with the following exposure variables: ALM index, grip strength, gait speed and age as linear terms. Contour plots were estimated at the mean age of the sex-pooled analysis sample

The probability of mortality within 5-years of follow-up (at the mean age of the analysis sample) according to ALM index, grip strength and gait speed is presented in Fig. 1. The difference in this risk of mortality at varying levels of ALM index was much smaller than the difference according to varying levels of grip strength and gait speed, supporting the lack of association between ALM index and mortality described above.

### Discriminative capacity for mortality

Discriminative capacity of the models for mortality, assessed using the C-index, is shown in Table 3. When included in univariate models, C-indices (95% CI) were greater for gait speed (0.61 (0.60, 0.61)) compared to grip strength (0.58 (0.57, 0.58)) and ALM index (0.53 (0.53, 0.54)); a C-index of 0.61 (0.61, 0.62) was also achieved when these three sarcopenia components were included in the same model. Including age and sex as exposures, along with ALM index, grip strength and gait speed increased the C-index from 0.61 (0.61, 0.62) to 0.67 (0.67, 0.68); additionally including key clinical risk factors (BMI, smoking status, alcohol consumption, fracture history and femoral neck BMD T-score) increased this value to 0.68 (0.67,0.69); and also including physical activity, ethnicity, education, fall history, self-rated health, cognitive function and number of comorbidities increased the C-index to 0.70 (0.69, 0.70).

Although grip strength and gait speed remained independently associated with mortality in the fully-adjusted model, only minimal improvement in the discriminative capacity for death was observed with the inclusion of the three sarcopenia components as exposures over and above the covariates (Table 3). For example, the C-index of the model with age and sex as exposures increased only from 0.65 (0.64, 0.66) to 0.67 (0.67, 0.68) when ALM index, grip strength and gait speed were also included. Similarly, the model with age, sex, BMI, smoking status, alcohol consumption, fracture history and femoral neck BMD T-score as exposures had a C-index of 0.66 (0.65, 0.67); this only increased to 0.68 (0.67, 0.69) with the inclusion of the three sarcopenia components. Finally, the C-index of the full model (age, sex, BMI, smoking status, alcohol consumption, fracture history, femoral neck BMD T-score, physical activity, ethnicity, education, fall history, self-rated health, cognitive function and number of comorbidities as covariates) only increased from 0.69 (0.68, 0.70) to 0.70 (0.69, 0.70) with the inclusion of ALM index, grip strength and gait speed.

Age- and sex-adjusted associations and mutually-adjusted associations between exposures and mortality risk are presented in Table 4, along with the C-indices of the models fitted. The majority of the exposures considered were independently associated with mortality after adjustment for age and sex and in mutually-adjusted analysis. However, a minimal model, including only age, sex, smoking status, education, self-rated health and number of comorbidities as covariates, achieved a C-index of 0.69 (0.68, 0.69), close to that of the mutually-adjusted model (0.70 (0.69, 0.70)) which comprised 14 exposures. Even though the increases in the C-indices reported in this section were modest, they were all statistically significant (*p* < 0.01), probably due to the large number of participants included in the analysis. All the C-index values reported were significantly different from 0.50 (*p* < 0.001).

**Table 4:** Mortality associations and discriminative capacity of individual exposures and combinations of exposures

Exposure	Age and sex included in all models			Mutually-adjusted model			Minimal model		
	Hazard ratio (95% CI)	*P* value	C-index (95% CI)	Hazard ratio (95% CI)	*P* value	C-index (95% CI)	Hazard ratio (95% CI)	*P* value	C-index (95% CI)
ALM index	1.01 (0.98,1.05)	0.520	0.65 (0.64,0.66)			0.70 (0.69,0.70)			0.69 (0.68,0.69)
Grip strength	1.24 (1.20,1.29)	< 0.001	0.66 (0.65,0.67)	1.15 (1.10,1.19)	< 0.001				
Gait speed	1.35 (1.30,1.39)	< 0.001	0.67 (0.66,0.68)	1.21 (1.17,1.26)	< 0.001				
Age	1.79 (1.75,1.84)	< 0.001	0.65 (0.64,0.66)	1.63 (1.58,1.68)	< 0.001		1.78 (1.73,1.83)	< 0.001	
Sex (female)	0.70 (0.64,0.78)	< 0.001	0.65 (0.64,0.66)	0.50 (0.44,0.56)	< 0.001		0.67 (0.61,0.74)	< 0.001	
BMI	1.05 (1.02,1.08)	0.002	0.65 (0.65,0.66)	0.98 (0.95,1.01)	0.156				
Current smoker	1.91 (1.71,2.13)	< 0.001	0.66 (0.65,0.67)	1.81 (1.61,2.02)	< 0.001		1.93 (1.73,2.16)	< 0.001	
High alcohol consumption	1.01 (0.94,1.09)	0.761	0.65 (0.64,0.66)						
Previous fracture	1.09 (1.02,1.16)	0.010	0.65 (0.64,0.66)	1.03 (0.97,1.10)	0.324				
Femoral neck BMD T-score	0.99 (0.97,1.02)	0.615	0.65 (0.64,0.66)						
Physical activity	0.89 (0.86,0.91)	< 0.001	0.66 (0.65,0.66)	0.96 (0.93,0.99)	0.004				
Ethnicity (BAME)	1.14 (1.06,1.23)	< 0.001	0.65 (0.65,0.66)	0.91 (0.84,0.99)	0.025				
Left school early	1.33 (1.22,1.44)	< 0.001	0.66 (0.65,0.66)	1.08 (0.99,1.18)	0.082		1.18 (1.09,1.29)	< 0.001	
Fall in previous year	1.11 (1.04,1.19)	0.001	0.65 (0.65,0.66)	1.00 (0.94,1.07)	0.889				
Self-rated health (< good)	1.86 (1.73,2.00)	< 0.001	0.67 (0.66,0.67)	1.39 (1.29,1.50)	< 0.001		1.54 (1.43,1.66)	< 0.001	
Low cognitive function	1.71 (1.52,1.92)	< 0.001	0.66 (0.65,0.67)	1.44 (1.27,1.64)	< 0.001				
Number of comorbidities	1.22 (1.20,1.25)	< 0.001	0.67 (0.66,0.68)	1.16 (1.13,1.18)	< 0.001		1.19 (1.16,1.21)	< 0.001	

Hazard ratios shown per SD lower level of ALM index, grip strength and gait speed; hazard ratios per SD higher level shown for other continuous exposures

*C-index* Harrell’s Concordance Index, *MrOS* Osteoporotic Fractures in Men Study, *Health ABC* Health, Aging and Body Composition Study, *HCS* Hertfordshire Cohort Study, *BAME* Black, Asian and minority ethnic, *ALM* Appendicular lean mass

High alcohol consumption: MrOS US (> 7 drinks per week); Health ABC (> 1 drink per day); HCS (> 14 units per week)

Physical activity: MrOS US (Physical Activity Scale for the Elderly score); Health ABC (Mcal/week); HCS (mins/day)

Left school early: MrOS US (did not complete high school); Health ABC (did not complete Grade 12); HCS (left school before age 15 years)

Previous fracture: MrOS US (fracture since age 50 years); fracture since age 45 for Health ABC and HCS

Low cognitive function: MrOS US and Health ABC (Modified Mini-Mental State Exam score < 80); HCS (Mini-Mental State Exam score < 24)

Number of comorbidities out of: heart disease, lung disease, hypertension, diabetes, stroke, arthritis, osteoporosis, thyroid disease, Parkinson's and cancer

### Sensitivity analyses

Supplementary Tables 2–4 present results from the Cox regression analyses when conducted within each cohort. These results were broadly similar to those from the main analysis where cohorts were pooled together and a shared statistical frailty factor was included in Cox models to account for differences in the underlying mortality risk between cohorts.

## Discussion

In this study, lower grip strength and gait speed were independently associated with mortality after accounting for a range of sociodemographic, lifestyle and clinical factors. In contrast, the association for ALM index was weaker in magnitude and not different from the null after consideration of other characteristics. However, in multivariate models, it was apparent that grip strength and gait speed did not substantially add predictive value for death over and above age and sex, and only minimally above models including clinical risk factors, educational factors and comorbidity burden. Thus, a model including only age, sex, smoking status, education, self-rated health and comorbidity burden as covariates achieved a similar predictive performance regarding mortality as a mutually-adjusted model comprising 14 individual exposures.

Many studies have examined grip strength, gait speed and lean mass measures in relation to risk of adverse health outcomes and have established stronger associations regarding grip strength and gait speed in comparison with measures of lean mass [6, 8, 20]. Indeed, the importance of physical performance as a predictor of mortality in older people has been reported previously [21]. However, research comparing the predictive or discriminative capacity of individual sarcopenia components in relation to mortality is limited. A study involving 645 US haemodialysis patients evaluated four lean mass indices, grip strength, and gait speed for their predictive accuracy for mortality [22]. The base Cox model, which included age, sex, ethnicity and comorbidities, had a C-index of 0.63. This increased proportionally by 5% to 0.66 with the addition of gait speed only, and by a further 3% to 0.68 with the addition of grip strength only. However, none of the lean mass indices achieved C-indices greater than 0.65 when individually added to the base model. This supports our study’s findings of higher C-indices for grip strength (0.58 (95% CI: 0.57, 0.58)) and gait speed (0.61 (0.60, 0.61)) as univariate exposures, compared to ALM index (0.53 (0.53, 0.54)). However, larger increases in C-indices were observed with the inclusion of grip strength and gait speed compared to our study, where the inclusion of all sarcopenia components only increased the C-index of the model with age and sex as exposures from 0.65 (0.64, 0.66) to 0.67 (0.67, 0.68). This could be due to the significantly younger average age of 56.7 years for the haemodialysis patients compared to 73.8 years in our study, leading to age having a higher discriminative capacity for mortality in our study and thus less improvement in predictive accuracy from additionally including the sarcopenia components. Differences in findings could also be due to differences in the study setting (haemodialysis patients versus community-dwelling older participants in our study).

There are several potential biological mechanisms which might underpin the observed associations between lower grip strength and gait speed and increased mortality risk. These may represent common underlying mechanisms for both exposure and outcome or more directly from the muscle measures to mortality. For example, underlying physiological processes such as age-related chronic inflammation (inflammaging), oxidative stress, accumulation of senescent cells, and endocrine dysfunction might be causally linked to declines in grip strength and gait speed, as well as increased mortality risk [23]. Furthermore, independent walking requires not only sufficient strength but also adequate motor control, balance, and coordination. It involves multiple anatomical systems, including the respiratory, cardiovascular, and nervous systems. Consequently, slower walking speed might indicate impairments in these systems, leading to a higher mortality risk [19].

As well as increasing risk of mortality, sarcopenia also has a significant impact on the health of older people more widely, affecting both quality of life and daily functionality. For example, in a recent systematic review and meta-analysis, investigating the impact of age-related sarcopenia on health-related quality of life using the Sarcopenia Quality of Life (SarQoL) questionnaire, individuals with sarcopenia had significantly lower health-related quality of life compared to those without sarcopenia [3]. Furthermore, in another systematic review and meta-analysis, sarcopenia was found to be associated with increased risk of fractures and falls, which can result in physical disability and loss of independence [24]. Reduced muscle strength and function are key components of sarcopenia and may limit the ability to perform daily activities such as walking, climbing stairs, and carrying groceries, thereby reducing individual autonomy and self-sufficiency. Indeed, sarcopenia was associated with increased risk of disability regarding basic activities of daily living (odds ratio: 1.58, 95%CI: 1.18–2.11) and instrumental activities of daily living (1.87, 95%CI: 1.40–2.51)) among participants, aged 60 years and older, from the China Health and Retirement Longitudinal Study [25].

There are several possible reasons why including additional exposures, especially sarcopenia components, provided limited improvement in mortality prediction in these cohorts over and above other factors. Socioeconomic disadvantage, poor health behaviours and greater comorbidity are risk factors for low grip strength and gait speed [26]. Therefore, sarcopenia components are likely to be correlated with these factors so the additional information on mortality risk provided by sarcopenia components may be limited if information on these other factors is already available. Similarly, exposures such as comorbidity burden and self-rated health are on the causal pathway from lifestyle factors to adverse health events and are known to be correlated with lifestyle factors [27, 28]. Therefore, improvements in discriminative capacity from the incorporation of lifestyle factors in a model already including comorbidity and self-rated health as exposures may be limited. Indeed, understanding the temporal and causal relationships between sarcopenia components, other participant characteristics and risk of mortality is a worthwhile topic for future research, but this would require longitudinal data ascertained over multiple time-points. Finally, participants of the Health ABC cohort had no mobility disability at baseline, and MrOS participants had to be able to walk without the assistance of another person. Therefore, measures such as grip strength and gait speed may have less variation in these two cohorts compared to in the general population of this age range, resulting in these measures having lower discriminative capacity regarding mortality. Furthermore, gait speed declines with age substantially [29], resulting in a strong correlation between older age and slower gait speed. Therefore, the improvement in discriminative capacity of gait speed regarding mortality over and above age may be minimal.

Strengths of this study are that analyses were based on a large number of community-dwelling participants and that participants were recruited from cohorts where data were rigorously collected according to strict protocols. However, this study does have some limitations. The higher physical capability levels of the Health ABC and MrOS cohort, as discussed previously, may limit the generalizability of findings to the wider population of older people in this age group. Furthermore, the exclusion of participants with higher levels of disability from this study, such as nursing home residents or individuals with advanced disability, suggests that that the typical values of grip strength, gait speed and ALM index, and the risk of mortality in these studies are likely to differ compared to that of the general population in this age range. The generalizability of findings may also be limited by the fact that white men in the MrOS Study comprised 59% of the analysis sample; only 17% were women and 19% were BAME. This suggests that the findings of this study would be most applicable to older community-dwelling Caucasian men, who are able to walk unaided, and may be less generalisable to older women and older BAME individuals, and those in poorer health such as nursing home residents. Third, DXA lean mass is only a surrogate measure of muscle mass and also includes organ weight, water and other non-fat and non-bone soft tissue; other techniques such as the D_3_-Creatine (D3-Cr) dilution method, may provide a more direct and accurate assessment of muscle mass according to previous publications [30–32]. Computed tomography (CT) and magnetic resonance imaging (MRI) have also been shown to be useful in relating muscle measures to clinical outcomes. For example, adverse muscle composition (low muscle volume and high muscle fat infiltration), ascertained using MRI, was independently related to increased mortality risk in the UK Biobank [33], and CT-derived lower baseline muscle area was associated with increased risk of mortality in the Health ABC Study [34]. Fourth, functional status, arguably a more relevant outcome for sarcopenia than mortality, was not considered as an outcome in this study. This is because, unlike mortality, functional status was defined differently across the cohorts considered. Indeed, in a study of older outpatients admitted to a tertiary health centre, probable sarcopenia (low grip strength) was associated with subsequent deterioration in functional status [35]. However, this was only the case for population-specific thresholds, rather than the EWGSOP2 grip strength threshold. Fifth, the following limitations of Harrell’s Concordance Index in a survival analysis setting have been reported: it only depends on the ranks of the predicted probabilities; it can be insensitive to the addition of statistically and clinically significant exposures; it is strongly affected by the censoring distribution; and the ability to identify the difference in risk between any two subjects is often not of clinical interest [36]. However, the C-index was selected over other indices such as the Brier score due to its wide use in survival analysis, ease of interpretation, and focus on discriminative capacity. Furthermore, estimated 5-year cumulative incidence functions for mortality demonstrated minimal differences in mortality risk according to quartiles of grip strength and gait speed after controlling for the other risk factors considered (data not shown). This supports the lack of improvement in discriminative capacity of grip strength and gait speed, over and above other risk factors, even regarding short term mortality. Sixth, we cannot exclude the possibility that residual confounding could have contributed to the associations observed. For example, the inability to harmonize the required variables between cohorts meant that it was not possible to include mental health factors, such as measures of social isolation, loneliness and depression, as potential confounders in this study. Finally, some assessment protocols differed between cohorts, such as the distance used for gait speed tests that were 8ft in HCS and 6 m in the other cohorts. However, findings were similar when HCS gait speed values were transformed to those expected over 6 m using published equations [18, 19]. Furthermore, differences in the following between cohorts may have affected the analysis when the cohorts were pooled together: lack of calibration of DXA and grip strength devices between cohorts; methods used to ascertain exposures; and underlying risk of mortality. However, Cox models with cohort as the shared statistical frailty factor were implemented to account for differences in the underlying mortality risk between cohorts, and findings were broadly similar when replicated internally within each cohort.

Whilst a non-specific approach to predicting mortality risk may have limited clinical utility, insomuch as it does not point to any particular remedial intervention, greater risk of death is, de facto, likely to indicate poorer health, and thus identify individuals who may benefit from further clinical assessment. This might include specific physical measures relating to muscle strength and mobility, for which interventions such as exercise regimens might be appropriate [37–39]. However, in the first instance, given that the adjunctive risk information provided by such measurements appears to be minimal, any approach to high-level assessment of mortality risk may be most appropriately predicated on information likely to be already available in the primary and/or secondary care record rather than on further measures. Indeed, the modest set of clinical risk factors considered in Set 2 corresponds to input variables incorporated in the FRAX^®^ Fracture Risk Assessment Tool [40], suggesting that this algorithm might be further evaluated in this context.

In conclusion, we have demonstrated that grip strength and gait speed are modest predictors of mortality during follow-up in three international cohorts, with minimal mortality association for DXA ALM index. Whilst lower grip strength and gait speed retained an association with greater mortality risk after adjustment for a wide range of covariates, the improvement in risk prediction gained through the addition of grip strength and gait speed to risk factor-based models was minimal. This suggests that approaches to clinical status based on mortality might most usefully incorporate existing measures likely to be readily available from the clinical record, rather than undertaking new assessments of muscle-related measures such as grip strength, gait speed and ALM index.

### Supplementary Information

Below is the link to the electronic supplementary material.

Supplementary file1 (XLSX 20 KB)

Supplementary file2 (DOCX 30 KB)

## Data Availability

The data used in this study cannot be shared due to consent restrictions.
