# Supplementary material for: Predictive value of sarcopenia components for all-cause mortality: findings from population-based cohorts
Source: Aging Clin Exp Res. 2024 Jun 6;36(1):126. doi: 10.1007/s40520-024-02783-x (PMC11156728; doi:10.1007/s40520-024-02783-x)
Supplement: Supplementary file 2 — Supplementary file2 (DOCX 30 KB) [file 40520_2024_2783_MOESM2_ESM.docx]

**Article title:** Predictive value of sarcopenia components for all-cause mortality: findings from population-based cohorts

**Journal name:** Aging Clinical and Experimental Research

**Author names:** Leo D Westbury, Nicholas C Harvey, Charlotte Beaudart, Olivier Bruyère, Jane A Cauley, Peggy Cawthon, Alfonso J Cruz-Jentoft, Elizabeth M Curtis, Kristine Ensrud, Roger A Fielding, Helena Johansson, John A Kanis, Magnus K Karlsson, Nancy E Lane, Laetitia Lengelé, Mattias Lorentzon, Eugene McCloskey, Dan Mellström, Anne B Newman, Claes Ohlsson, Eric Orwoll, Jean-Yves Reginster, Eva Ribom, Björn E Rosengren, John T. Schousboe, Elaine M Dennison, Cyrus Cooper and the International Musculoskeletal Ageing Network

**Affiliation and e-mail address of the corresponding author:**

Prof Nicholas C. Harvey

MRC Lifecourse Epidemiology Centre

University of Southampton

Southampton

UK

Tel: 02380 777624; Email: [nch@mrc.soton.ac.uk](mailto:nch@mrc.soton.ac.uk)

| **Supplementary Table 2: Mortality associations for sarcopenia components and discriminative capacity of models, depending on exposures included, among participants of the Osteoporotic Fractures in Men (MrOS) Study (USA)** | | | | | | | |
| --- | --- | --- | --- | --- | --- | --- | --- |
|  |  |  |  |  |  |  |  |
| **Exposures included** | **C-index**  **(95% CI)** | **Associations for sarcopenia components (per SD lower level of component)** | | | | | |
|  |  | **ALM index (z-score)** | | **Grip strength (z-score)** | | **Gait speed (z-score)** | |
|  |  | **HR (95% CI)** | **P-value** | **HR (95% CI)** | **P-value** | **HR (95% CI)** | **P-value** |
| ALM index | 0.56 (0.55,0.57) | 1.24 (1.19,1.30) | <0.001 | - | - | - | - |
| Grip strength | 0.62 (0.61,0.63) | - | - | 1.67 (1.60,1.74) | <0.001 | - | - |
| Gait speed | 0.62 (0.61,0.63) | - | - | - | - | 1.58 (1.52,1.64) | <0.001 |
| ALM index, grip strength | 0.62 (0.61,0.63) | 1.08 (1.03,1.13) | <0.001 | 1.64 (1.57,1.71) | <0.001 | - | - |
| ALM index, gait speed | 0.63 (0.62,0.64) | 1.24 (1.19,1.30) | <0.001 | - | - | 1.59 (1.53,1.65) | <0.001 |
| Grip strength, gait speed | 0.65 (0.64,0.66) | - | - | 1.51 (1.45,1.58) | <0.001 | 1.44 (1.39,1.50) | <0.001 |
| ALM index, grip strength, gait speed | 0.65 (0.64,0.66) | 1.12 (1.07,1.17) | <0.001 | 1.46 (1.39,1.53) | <0.001 | 1.46 (1.40,1.51) | <0.001 |
| ALM index, grip strength, gait speed, Set 1 | 0.70 (0.69,0.71) | 0.99 (0.94,1.03) | 0.503 | 1.22 (1.17,1.28) | <0.001 | 1.28 (1.23,1.33) | <0.001 |
| ALM index, grip strength, gait speed, Set 2 | 0.70 (0.69,0.71) | 1.08 (1.01,1.15) | 0.019 | 1.20 (1.14,1.26) | <0.001 | 1.26 (1.21,1.31) | <0.001 |
| ALM index, grip strength, gait speed, Set 3 | 0.72 (0.71,0.73) | 1.03 (0.96,1.10) | 0.404 | 1.16 (1.11,1.22) | <0.001 | 1.18 (1.13,1.23) | <0.001 |
| Set 1 | 0.68 (0.67,0.69) | - | - | - | - | - | - |
| Set 2 | 0.69 (0.68,0.70) | - | - | - | - | - | - |
| Set 3 | 0.71 (0.70,0.72) | - | - | - | - | - | - |
| HR: Hazard ratio | | | | | | | |
| C-index: Harrell’s Concordance Index | | | | | | | |
| Exposures included in each adjustment set: | | | | | | | |
| Set 1: Age | | | | | | | |
| Set 2: Set 1, BMI, current smoker (yes/no), high alcohol consumption (yes/no), fracture since age 45 years (50 years in MrOS US Study) (yes/no), femoral neck BMD T-score | | | | | | | |
| Set 3: Set 2, physical activity, BAME ethnicity (yes/no), left school early (yes/no), fall in previous 12 months (yes/no), self-rated health of less than good (yes/no), low cognitive function (yes/no), number of comorbidities | | | | | | | |

| **Supplementary Table 3: Mortality associations for sarcopenia components and discriminative capacity of models, depending on exposures included, among participants of the Health, Aging and Body Composition (Health ABC) Study** | | | | | | | |
| --- | --- | --- | --- | --- | --- | --- | --- |
|  |  |  |  |  |  |  |  |
| **Exposures included** | **C-index**  **(95% CI)** | **Associations for sarcopenia components (per SD lower level of component)** | | | | | |
|  |  | **ALM index (z-score)** | | **Grip strength (z-score)** | | **Gait speed (z-score)** | |
|  |  | **HR (95% CI)** | **P-value** | **HR (95% CI)** | **P-value** | **HR (95% CI)** | **P-value** |
| ALM index | 0.52 (0.51,0.54) | 0.95 (0.91,0.99) | 0.022 | - | - | - | - |
| Grip strength | 0.52 (0.50,0.53) | - | - | 0.95 (0.91,1.00) | 0.041 | - | - |
| Gait speed | 0.57 (0.56,0.59) | - | - | - | - | 1.34 (1.26,1.41) | <0.001 |
| ALM index, grip strength | 0.52 (0.51,0.54) | 0.96 (0.91,1.02) | 0.183 | 0.98 (0.92,1.03) | 0.409 | - | - |
| ALM index, gait speed | 0.58 (0.56,0.59) | 0.95 (0.91,0.99) | 0.019 | - | - | 1.34 (1.26,1.41) | <0.001 |
| Grip strength, gait speed | 0.58 (0.57,0.59) | - | - | 0.89 (0.85,0.93) | <0.001 | 1.38 (1.30,1.46) | <0.001 |
| ALM index, grip strength, gait speed | 0.58 (0.57,0.59) | 1.02 (0.96,1.07) | 0.585 | 0.88 (0.83,0.94) | <0.001 | 1.38 (1.31,1.47) | <0.001 |
| ALM index, grip strength, gait speed, Set 1 | 0.63 (0.61,0.64) | 1.07 (1.01,1.13) | 0.025 | 1.09 (1.01,1.18) | 0.022 | 1.41 (1.33,1.50) | <0.001 |
| ALM index, grip strength, gait speed, Set 2 | 0.64 (0.63,0.65) | 0.96 (0.87,1.05) | 0.359 | 1.14 (1.05,1.23) | 0.002 | 1.38 (1.30,1.47) | <0.001 |
| ALM index, grip strength, gait speed, Set 3 | 0.66 (0.64,0.67) | 0.97 (0.88,1.08) | 0.640 | 1.10 (1.02,1.20) | 0.014 | 1.28 (1.20,1.37) | <0.001 |
| Set 1 | 0.59 (0.58,0.60) | - | - | - | - | - | - |
| Set 2 | 0.61 (0.59,0.62) | - | - | - | - | - | - |
| Set 3 | 0.64 (0.63,0.66) | - | - | - | - | - | - |
| HR: Hazard ratio | | | | | | | |
| C-index: Harrell’s Concordance Index | | | | | | | |
| Set 1: Age, sex | | | | | | | |
| Set 2: Set 1, BMI, current smoker (yes/no), high alcohol consumption (yes/no), fracture since age 45 years (50 years in MrOS US Study) (yes/no), femoral neck BMD T-score | | | | | | | |
| Set 3: Set 2, physical activity, BAME ethnicity (yes/no), left school early (yes/no), fall in previous 12 months (yes/no), self-rated health of less than good (yes/no), low cognitive function (yes/no), number of comorbidities | | | | | | | |

| **Supplementary Table 4: Mortality associations for sarcopenia components and discriminative capacity of models, depending on exposures included, among participants of the Hertfordshire Cohort Study** | | | | | | | |
| --- | --- | --- | --- | --- | --- | --- | --- |
|  |  |  |  |  |  |  |  |
| **Exposures included** | **C-index**  **(95% CI)** | **Associations for sarcopenia components (per SD lower level of component)** | | | | | |
|  |  | **ALM index (z-score)** | | **Grip strength (z-score)** | | **Gait speed (z-score)** | |
|  |  | **HR (95% CI)** | **P-value** | **HR (95% CI)** | **P-value** | **HR (95% CI)** | **P-value** |
| ALM index | 0.52 (0.44,0.60) | 0.94 (0.67,1.32) | 0.721 | - | - | - | - |
| Grip strength | 0.52 (0.43,0.62) | - | - | 1.08 (0.77,1.51) | 0.654 | - | - |
| Gait speed | 0.67 (0.58,0.76) | - | - | - | - | 2.49 (1.60,3.86) | <0.001 |
| ALM index, grip strength | 0.56 (0.46,0.65) | 0.84 (0.54,1.28) | 0.409 | 1.21 (0.79,1.84) | 0.383 | - | - |
| ALM index, gait speed | 0.67 (0.58,0.76) | 0.92 (0.66,1.29) | 0.624 | - | - | 2.50 (1.61,3.87) | <0.001 |
| Grip strength, gait speed | 0.66 (0.57,0.75) | - | - | 0.88 (0.62,1.24) | 0.453 | 2.64 (1.65,4.22) | <0.001 |
| ALM index, grip strength, gait speed | 0.66 (0.57,0.75) | 0.99 (0.65,1.53) | 0.977 | 0.88 (0.57,1.37) | 0.570 | 2.64 (1.63,4.26) | <0.001 |
| ALM index, grip strength, gait speed, Set 1 | 0.69 (0.60,0.78) | 1.31 (0.77,2.21) | 0.316 | 1.18 (0.72,1.95) | 0.507 | 2.48 (1.54,4.00) | <0.001 |
| ALM index, grip strength, gait speed, Set 2 | 0.76 (0.68,0.83) | 0.98 (0.46,2.08) | 0.951 | 1.34 (0.80,2.24) | 0.264 | 2.52 (1.53,4.15) | <0.001 |
| ALM index, grip strength, gait speed, Set 3 | 0.77 (0.70,0.85) | 0.89 (0.40,1.97) | 0.778 | 1.36 (0.77,2.38) | 0.289 | 2.26 (1.29,3.97) | 0.004 |
| Set 1 | 0.61 (0.51,0.70) | - | - | - | - | - | - |
| Set 2 | 0.68 (0.60,0.76) | - | - | - | - | - | - |
| Set 3 | 0.73 (0.65,0.81) | - | - | - | - | - | - |
| HR: Hazard ratio | | | | | | | |
| C-index: Harrell’s Concordance Index | | | | | | | |
| Set 1: Age, sex | | | | | | | |
| Set 2: Set 1, BMI, current smoker (yes/no), high alcohol consumption (yes/no), fracture since age 45 years (50 years in MrOS US Study) (yes/no), femoral neck BMD T-score | | | | | | | |
| Set 3: Set 2, physical activity, left school early (yes/no), fall in previous 12 months (yes/no), self-rated health of less than good (yes/no), low cognitive function (yes/no), number of comorbidities | | | | | | | |
